# Layer-specific changes of KCC2 and NKCC1 in the mouse dentate gyrus after entorhinal denervation

**DOI:** 10.3389/fnmol.2023.1118746

**Published:** 2023-05-24

**Authors:** Domenico Del Turco, Mandy H. Paul, Jessica Schlaudraff, Julia Muellerleile, Fran Bozic, Mario Vuksic, Peter Jedlicka, Thomas Deller

**Affiliations:** ^1^Institute of Clinical Neuroanatomy, Neuroscience Center, Goethe University Frankfurt, Frankfurt, Germany; ^2^Croatian Institute for Brain Research, School of Medicine, University of Zagreb, Zagreb, Croatia; ^3^Faculty of Medicine, Justus-Liebig-University Giessen, Giessen, Germany

**Keywords:** hippocampus, laser microdissection, RT-qPCR, entorhinal lesion, cation-chloride cotransporters, dedifferentiation

## Abstract

The cation-chloride cotransporters KCC2 and NKCC1 regulate the intracellular Cl^−^ concentration and cell volume of neurons and/or glia. The Cl^−^ extruder KCC2 is expressed at higher levels than the Cl^−^ transporter NKCC1 in mature compared to immature neurons, accounting for the developmental shift from high to low Cl^−^ concentration and from depolarizing to hyperpolarizing currents through GABA-A receptors. Previous studies have shown that KCC2 expression is downregulated following central nervous system injury, returning neurons to a more excitable state, which can be pathological or adaptive. Here, we show that deafferentation of the dendritic segments of granule cells in the outer (oml) and middle (mml) molecular layer of the dentate gyrus via entorhinal denervation *in vivo* leads to cell-type- and layer-specific changes in the expression of KCC2 and NKCC1. Microarray analysis validated by reverse transcription-quantitative polymerase chain reaction revealed a significant decrease in *Kcc2* mRNA in the granule cell layer 7 days post-lesion. In contrast, *Nkcc1* mRNA was upregulated in the oml/mml at this time point. Immunostaining revealed a selective reduction in KCC2 protein expression in the denervated dendrites of granule cells and an increase in NKCC1 expression in reactive astrocytes in the oml/mml. The NKCC1 upregulation is likely related to the increased activity of astrocytes and/or microglia in the deafferented region, while the transient KCC2 downregulation in granule cells may be associated with denervation-induced spine loss, potentially also serving a homeostatic role via boosting GABAergic depolarization. Furthermore, the delayed KCC2 recovery might be involved in the subsequent compensatory spinogenesis.

## Introduction

The cation-chloride cotransporters play a key role in maintaining the intracellular Cl^−^ concentration (Kaila et al., 2014). The sodium-potassium-chloride (Na^+^-K^+^-Cl^−^) cotransporter 1 (NKCC1), which is expressed in neurons as well as astrocytes (Yan et al., 2001a), oligodendrocytes (Wang et al., 2003), and microglia (Tóth et al., 2022), relies on the plasmalemmal Na^+^ gradient to transport two Cl^−^ ions into the cell along with one Na^+^ and one K^+^ ion, whereas the neuron-specific (Payne et al., 1996) potassium-chloride (K^+^-Cl^−^) cotransporter 2 (KCC2) relies on the plasmalemmal K^+^ gradient to transport one Cl^−^ and one K^+^ ion out of the cell in an electroneutral manner (Kaila et al., 2014). While there have been conflicting results regarding the developmental expression of NKCC1 in neurons (Virtanen et al., 2020), there is evidence for a developmental increase in KCC2 expression in humans as well as in rats (Dzhala et al., 2005). The expression of the Cl^−^ extruder KCC2 is necessary for GABA_A_ receptor-mediated hyperpolarization, which explains why GABA has a depolarizing effect in neonatal rat neurons (Rivera et al., 1999). The clinical relevance of this developmental shift is supported by the observations that KCC2 and NKCC1 expression levels are altered in several neurodevelopmental disorders associated with epilepsy (Aronica et al., 2007) and that mutations in the *SLC12A5* gene, which encodes KCC2, are associated with autism spectrum disorder and schizophrenia (Merner et al., 2015), two disorders of neuronal development (see also Cherubini et al., 2022).

Changes in KCC2 or NKCC1 expression have also been observed in several different animal models of CNS and PNS injury: KCC2 protein or *Kcc2* mRNA is downregulated following peripheral nerve injury (Coull et al., 2003), spinal cord injury (Boulenguez et al., 2010), fluid percussion injury (Bonislawski et al., 2007), axotomy (Nabekura et al., 2002; Shulga et al., 2008), ischemia/stroke (Papp et al., 2008; Jaenisch et al., 2010), and deafferentation (Tighilet et al., 2016), while NKCC1 is upregulated following traumatic brain injury (Lu et al., 2008; Hui et al., 2016; Zhang et al., 2016), spinal cord injury (Yan et al., 2018) and ischemia (Yan et al., 2001b). However, so far, only general, spatially unresolved lesion-induced changes of KCC2 and NKCC1 have been studied. It has remained unclear whether a precise, anatomically well-defined lesion can evoke spatially restricted, layer-specific alterations in KCC2 or NKCC1 expression.

Therefore, we combined laser microdissection with microarray analysis and reverse transcription-quantitative polymerase chain reaction (RT-qPCR) to explore layer-specific changes in KCC2 and NKCC1 expression at different time points following entorhinal denervation. This approach has been widely used to study both the direct effects of axonal injury on the neurons in the entorhinal cortex as well as the secondary effects of the deafferentation on the neurons in the hippocampus, which are located far away from the lesion site and therefore unlikely to suffer direct damage (Deller and Frotscher, 1997; Perederiy and Westbrook, 2013). Moreover, the layered arrangement of the different synaptic inputs to the dentate gyrus granule cells allows one to differentiate denervated from non-denervated dendritic segments and analyze layer-specific morphological, molecular, and functional changes following entorhinal denervation (Deller et al., 2001, 2006). Our analysis revealed that entorhinal denervation induced layer-specific changes in the expression levels of KCC2 and NKCC1 in the dentate gyrus, which might reflect homeostatic adaptations to the loss of synaptic input.

## Materials and methods

### Entorhinal denervation *in vivo*

Unilateral transection of the left perforant path was performed using a wire knife (David Kopf Instruments) as described previously (Del Turco et al., 2003). Adult C57Bl6/J male mice (3–4 months old; Janvier) survived 1, 3, 7, 14 or 28 days after entorhinal denervation *in vivo*. Experiments were designed to analyze control and lesioned animals of the same age. All efforts were made to minimize the suffering of experimental animals. Animal care and experimental procedures were approved by the Regierungspräsidium Darmstadt (Germany) and by the Ethics Committee of the University of Zagreb School of Medicine (Croatia).

### Laser microdissection and RNA processing

Mouse brains were rapidly removed from the cranium, immediately flash-frozen in −70°C isopentane and stored at −80°C until further processing. For laser microdissection, 16 μm thin sections of the septal hippocampus were cut with a cryostat and mounted on polyethylene naphthalene (PEN) membrane slides (Leica Microsystems). After fixation in −20°C cold 75 and 100% ethanol, sections were stained quickly with 1% cresyl violet and dehydrated briefly in 75 and 100% ethanol. Using a Leica LMD6500 system (Leica Microsystems), defined tissue portions of the granule cell layer (gcl) and the outer (oml) and middle (mml) molecular layer of the dentate gyrus were collected separately from the same brain sections. Total RNA was isolated using the RNeasy Plus Micro Kit (Qiagen) according to the manufacturer’s recommendations. RNA integrity was analyzed using the Agilent 2,100 Bioanalyzer system with Agilent RNA 6000 Pico Kit (Agilent Technologies).

### Microarray analysis

Microarray was performed in the Microarray Facility Tübingen (Germany). First, total RNA from three animals per time point was amplified using the Ovation Pico WTA System (NuGEN). The Encore Biotin Module Kit (NuGEN) was used for the fragmentation and labeling of cDNA for further analysis. Finally, samples were hybridized using Affymetrix GeneChip Mouse Gene 1.0 ST arrays (Affymetrix). Gene expression data were analyzed using Affymetrix Expression Console software (Affymetrix) and Partek Genomics Suite 6.5 software (Partek). Data were normalized and filtered for transcripts, which were differentially expressed between lesioned and control animals. Significance with *p* ≤ 0.05 was calculated using analysis of variance (ANOVA).

### Reverse transcription-quantitative polymerase chain reaction analysis

Total RNA from three to four animals (control and 7 days post-lesion, dpl) was reversely transcribed using High-Capacity cDNA Reverse Transcription Reagents Kit (Applied Biosystems) following the manufacturer’s recommendations. cDNA was preamplified before quantitative polymerase chain reaction (qPCR) using TaqMan PreAmp Master Mix (Applied Biosystems). qPCR conditions were carried out using TaqMan Gene Expression Master Mix (Applied Biosystems), and amplification was performed using the StepOnePlus Real-Time PCR System (Applied Biosystems). In addition, qPCR products were checked using the Agilent 2,100 Bioanalyzer system with Agilent DNA 1000 Chips (Agilent Technologies) to verify product specificity and amplicon size of TaqMan assays (Table 1). No signals were detected in no-template controls. Primer efficiencies and quantification cycle (Cq) values were calculated using LinRegPCR Software (version 12.7) on amplification data exported from the StepOnePlus Software. A tissue-specific index of three reference genes (*Gapdh*, *Rpl13a* and *Sdha* for the oml/mml and *Gapdh*, *Hprt* and *Pgk1* for the gcl) was used for normalization. qPCR data were tested for statistical significance (*p* ≤ 0.05) using the two-tailed Student’s *t*-test.

**Table 1:** TaqMan assay information for RT-qPCR used in this study.

Gene symbol	Official full name (NCBI)	TaqMan-Assay (Applied Biosystems)	Size (bp)
*Kcc2*	Solute carrier family 12, member 5 *(Slc12a5)*	Mm00803929	92
*Nkcc1*	Solute carrier family 12, member 2 *(Slc12a2)*	Mm01265951	90
*Gapdh*	Glyceraldehyde-3-phosphate dehydrogenase	Mm99999915	107
*Hprt*	Hypoxanthine guanine phosphoribosyl transferase	Mm01318743	125
*Pgk1*	Phosphoglycerate kinase 1	Mm00435617	137
*Rpl13a*	Ribosomal protein L13A	Mm01612987	122
*Sdha*	Succinate dehydrogenase complex, subunit A	Mm01352366	82

### Immunofluorescence

Mice were deeply anesthetized with an overdose of pentobarbital (300 mg/kg body weight) and perfused with 0.9% NaCl followed by 4% paraformaldehyde (PFA) in phosphate-buffered saline (pH 7.4). Brains were removed, post-fixed overnight (o/n) in 4% PFA and sectioned in the coronal plane (40 μm) using a vibratome (VT1000 S, Leica). Free-floating sections containing the dorsal part of the hippocampus were incubated in a blocking buffer containing 0.3% Triton X-100 and 5% bovine serum albumin (BSA) in 0.05 M tris-buffered saline (TBS) for 30 min at room temperature followed by incubation in the primary antibody (diluted in 0.1% Triton X-100 and 1% BSA in 0.05 M TBS) for 2 days at room temperature (RT). After several washes, sections were incubated with the appropriate secondary Alexa-conjugated antibodies (1∶2000, Invitrogen) for 4 h at RT and finally mounted in Fluorescent Mounting Medium (Dako). Nuclei were counterstained with Draq5 (1∶10,000, eBioscience, 65–0880-92). The following primary antibodies were used for this study at the specified dilutions: mouse anti-glial fibrillary acidic protein (GFAP) (1∶500, Sigma-Aldrich), rabbit anti-KCC2 (1:1000, Merck, 07–432), and mouse anti-NeuN (1:1000, Chemicon, MAB377). For the detection of NKCC1, we used an affinity-purified goat polyclonal antibody raised against a peptide mapping near the N-terminus of NKCC1 (goat anti-NKCC1; 1:500, Santa Cruz, sc-21,545), which has been validated using NKCC1 knockout (−/−) mice (e.g., Mao et al., 2012; Jalali et al., 2017) and has been subsequently employed in a number of studies (e.g., Luo et al., 2018; Weng et al., 2018; Korrapati et al., 2019; Morell et al., 2020). Fluorescent images were acquired using confocal laser scanning microscopy (Eclipse C1 Plus, Nikon). The immunofluorescence signal was measured using Fiji, an open-source image processing package based on ImageJ.^1^ Briefly, multi-channel images were split into individual channels. Six regions of interest (ROIs) per layer (i.e., oml/mml and iml) ipsi- and contralaterally per animal (7 animals) were analyzed using the ‘ROI Manager’ function. All quantitative assessments were performed in a blinded manner. Data were analyzed using GraphPad Prism (GraphPad Software, United States). Statistical significance was determined using Kruskal-Wallis test with Dunn’s multiple comparison test, *p ≤ 0.05.

## Results

### Laser microdissection of the outer and middle molecular layer and granule cell layer of the dentate gyrus

**Figure 1:**
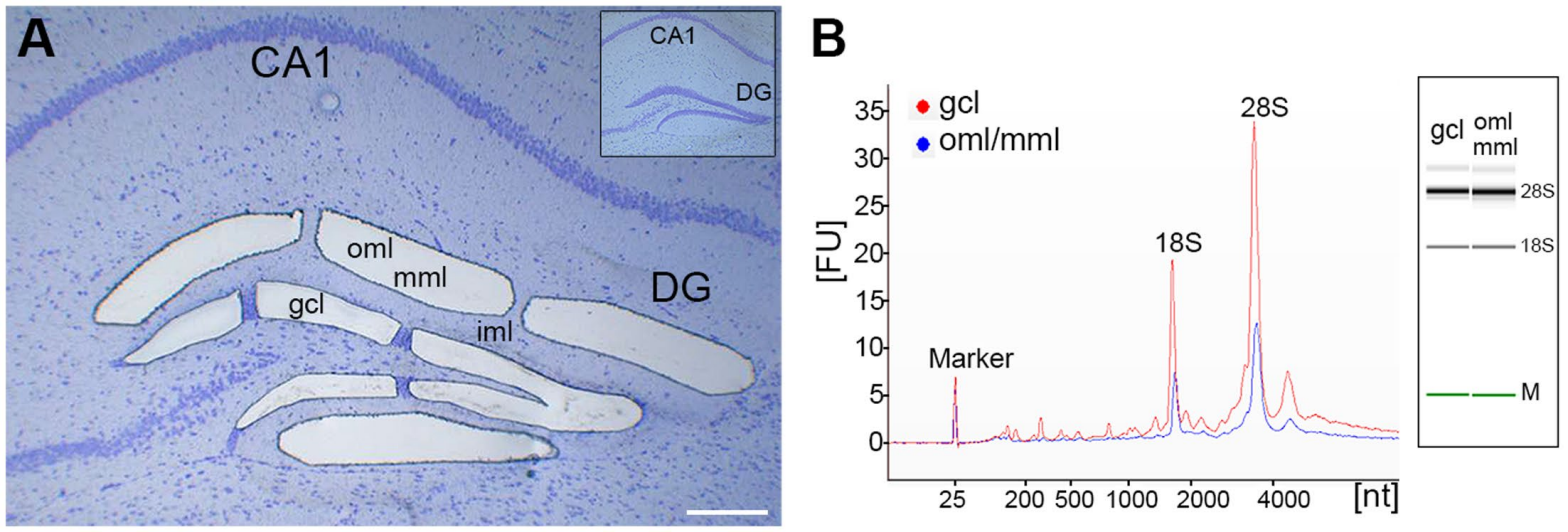
Laser microdissection of dentate layers. **(A)** Hippocampal section (coronal plane, dorsal part of the hippocampus, cresyl violet staining) before (inset) and after laser microdissection of the granule cell layer (gcl) and the outer (oml) and middle (mml) molecular layer of the dentate gyrus (DG). **(B)** RNA integrity analysis of total RNA isolated from the dissected gcl (red) and from the oml/mml (blue) demonstrating highly intact RNA (RIN-values: 8.15–8.3; Agilent 2,100 Bioanalyzer). CA1: hippocampal subfield (cornu ammonis region 1); FU, fluorescent units; nt, nucleotides; M, Marker. Scale bar: 200 μm.

To be able to perform later microarray and qPCR analyses of a layer-specific expression of KCC2 and NKCC1, laser microdissection was used to isolate separately the outer (oml) and middle (mml) molecular layer and the granule cell layer (gcl) of the dentate gyrus. For this approach, rapid histological staining was performed, and an ultraviolet laser was used to dissect the tissue in a contact-free environment, thereby ensuring the isolation of high-quality RNA for further processing. This method allowed us to dissect the oml/mml and the gcl from the same brain slices of control and lesioned animals, thus minimizing biological and technical variability in this study (Figure 1A). RNA integrity analysis of total RNA isolated from the oml/mml and gcl revealed highly intact RNA with RIN-values >8 (Figure 1B) as determined by the Agilent 2,100 Bioanalyzer system.

### Dentate layer-specific analysis of *Kcc2* mRNA and *Nkcc1* mRNA using microarray analysis and reverse transcription – quantitative PCR

Microarray analysis using Affymetrix GeneChip Mouse Gene 1.0 ST arrays was employed to study changes in the mRNA expression of the *K^+^-Cl^−^ cotransporter 2* (*Kcc2*) and the *Na^+^-K^+^-Cl^−^ cotransporter 1* (*Nkcc1*) in dentate regions, i.e., the oml/mml and gcl of the dentate gyrus, after entorhinal denervation. Laser-microdissected tissue from three animals per time point (control, 1, 3, 7, 14, 28 dpl) was used for microarray analysis. After entorhinal denervation, *Nkcc1* mRNA and *Kcc2* mRNA were found to be differentially expressed in the oml/mml compared to the gcl: *Nkcc1* mRNA showed a layer-specific upregulation only in the oml/mml (1.5-fold at 3 dpl, 1.4-fold at 7 dpl and 1.4-fold at 14 dpl), whereas *Kcc2* mRNA was downregulated specifically in the gcl (−1.7-fold at 1 dpl and − 3.4-fold at 7 dpl) (Table 2).

**Table 2:** Microarray analysis of *Kcc2* mRNA and *Nkcc1* mRNA in the outer/middle molecular layer and granule cell layer of the dentate gyrus after entorhinal denervation.

Gene	Probeset ID	Granule cell layer					Outer/middle molecular layer				
		1 dpl	3 dpl	7 dpl	14 dpl	28 dpl	1 dpl	3 dpl	7 dpl	14 dpl	28 dpl
		FC	FC	FC	FC	FC	FC	FC	FC	FC	FC
		Value of *p*	Value of *p*	Value of *p*	Value of *p*	Value of *p*	Value of *p*	Value of *p*	Value of *p*	Value of *p*	Value of *p*
***Kcc2***	10,478,647	**−1.6724**	−1.5839	**−3.3607**	−1.4793	−1.6023	−1.0011	−1.0248	−1.0726	−1.0004	1.2657
		**0.0332**	0.1237	**0.0015**	0.1677	0.1043	0.9955	0.9047	0.7595	0.9984	0.3149
***Nkcc1***	10,455,873	−1.6574	−1.9566	−1.0732	−1.664	−1.2002	1.2614	**1.53**	**1.4375**	**1.4249**	1.2219
		0.2593	0.2575	0.9015	0.366	0.7409	0.0649	**0.0035**	**0.0159**	**0.0101**	0.1406

Values in bold indicate significant changes (*p* ≤ 0.05, ANOVA) compared to unlesioned controls. dpl, days post-lesion; FC, fold change.

**Figure 2:**
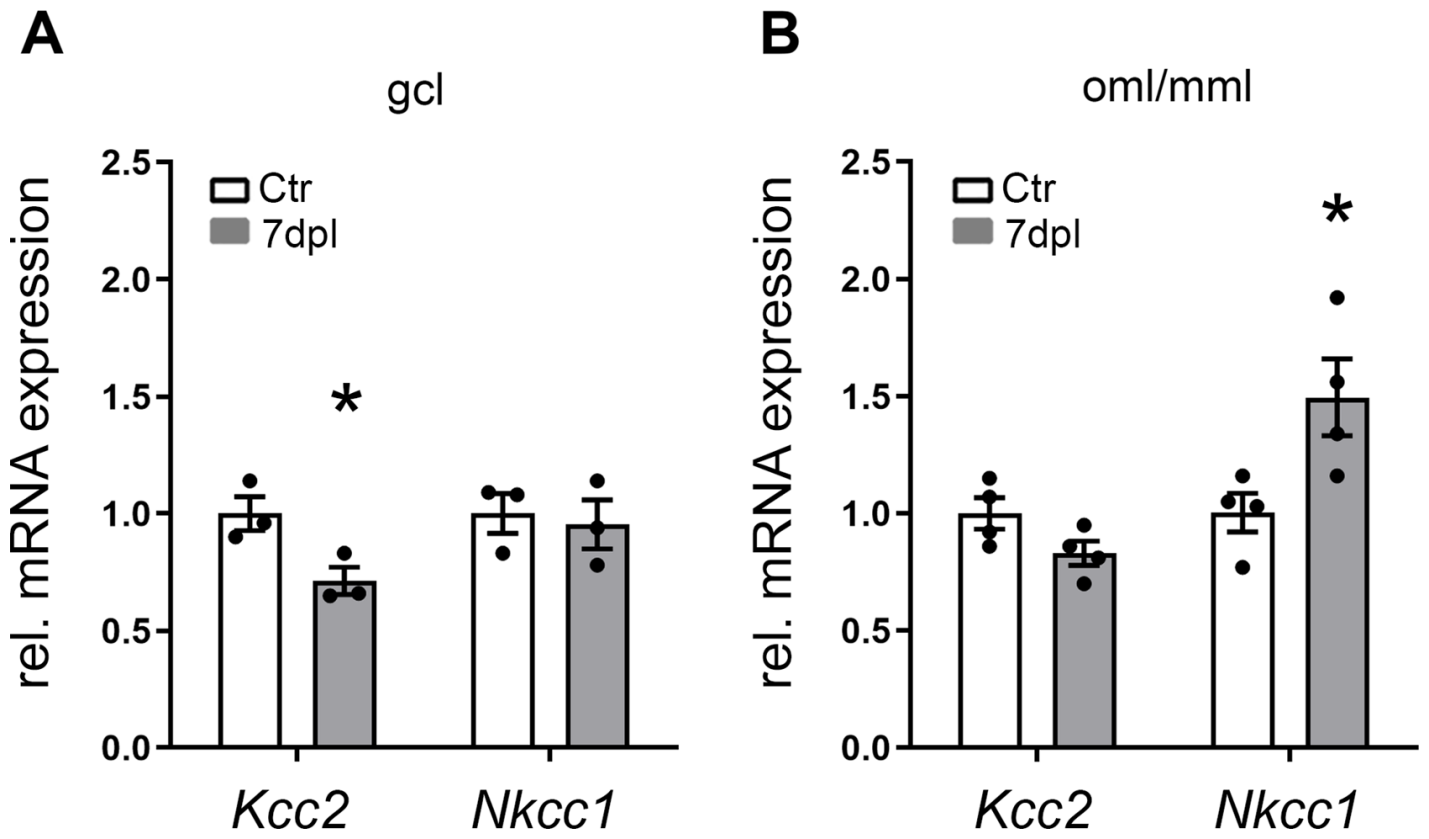
Layer-specific expression changes of *Kcc2* mRNA and *Nkcc1* mRNA in dentate layers following entorhinal denervation. RT-qPCR analysis for *Kcc2* mRNA and *Nkcc1* mRNA revealed layer-specific expression changes in the granule cell layer (gcl) **(A)** compared to the outer and middle molecular layer (oml/mml) **(B)** of the dentate gyrus 7 days post-lesion. *Kcc2* mRNA was found to be downregulated (−1.4-fold) only in the gcl, whereas *Nkcc1* mRNA was selectively upregulated (~1.5-fold) in the denervated oml/mml. *N* (animals) = 3–4; two-tailed Student’s *t*-test, *p ≤ 0.05.

Next, reverse transcription-quantitative PCR (RT-qPCR) analysis was performed for *Nkcc1* mRNA and *Kcc2* mRNA to validate lesion-associated changes in gene expression obtained by microarray analysis. As the greatest change in *Kcc2* mRNA expression was observed at 7 dpl, this time point was chosen for the RT-qPCR analysis. In the denervated oml/mml, upregulation of *Nkcc1* mRNA (∼1.5-fold) could be confirmed at 7 dpl, whereas no significant changes for *Kcc2* mRNA were found (Figure 2B). In contrast, *Kcc2* mRNA expression (−1.4-fold) was significantly downregulated in the denervated gcl at 7 dpl compared to the control. No change in *Nkcc1* mRNA expression was found in the gcl after the lesion (Figure 2A).

### Downregulation of KCC2 protein in the outer and middle molecular layer of the dentate gyrus after entorhinal denervation

**Figure 3:**
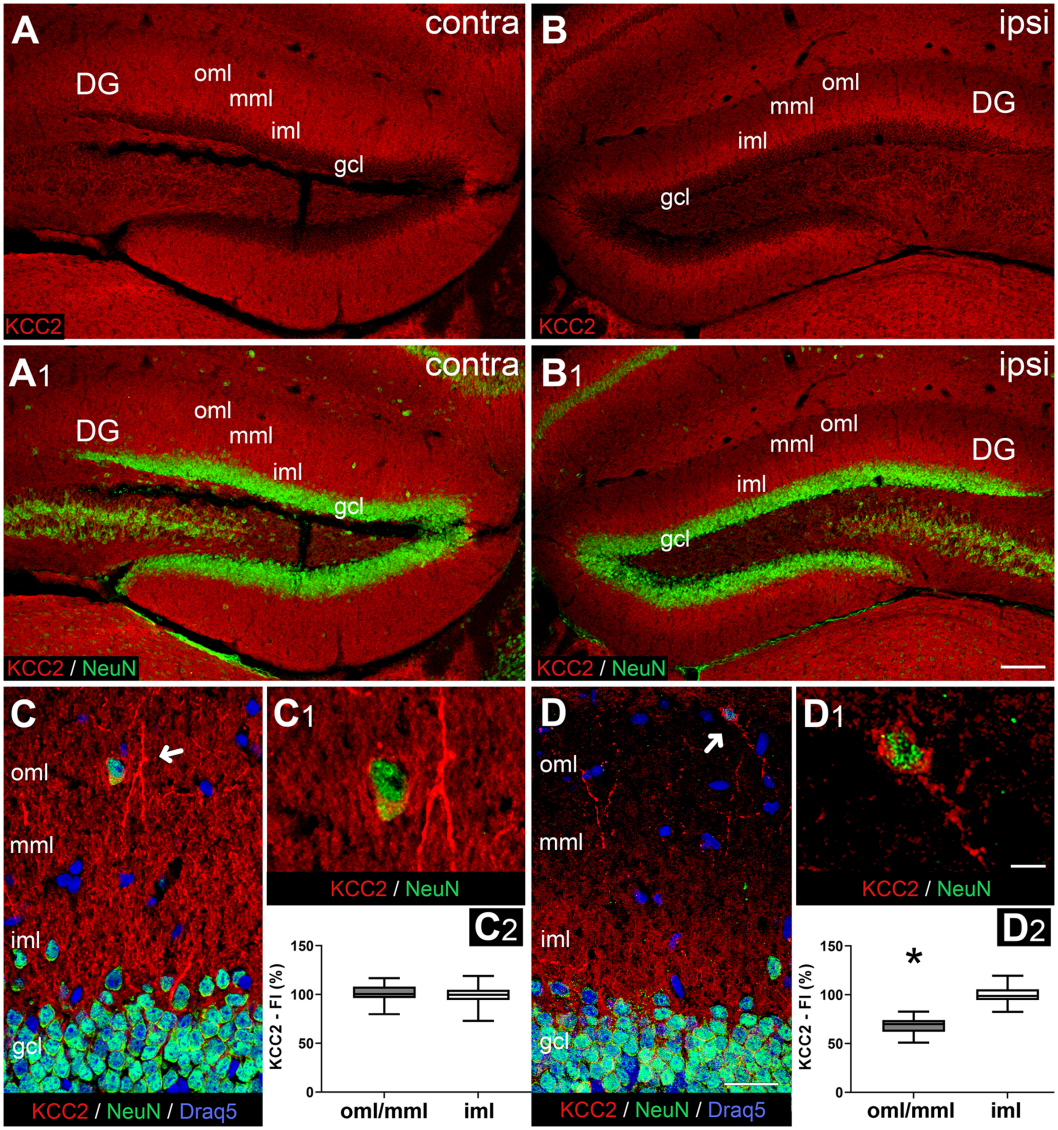
Decreased KCC2 protein expression in the outer and middle molecular layer of the dentate gyrus 7 days after entorhinal denervation. Immunofluorescence in combination with confocal analysis demonstrates decreased expression of KCC2 protein in the denervated outer (oml) and middle (mml) molecular layer of the dentate gyrus (DG). Control (contra): **(A,C)**; denervation (ipsi): **(B,D)**. Double-immunofluorescence for KCC2 (red) and the neuronal marker NeuN (green) demonstrates KCC2-positive neuronal cells (**C,D**: arrows), which could be found both in the non-denervated and denervated oml/mml; **(C1,D1)**: higher magnification of KCC2-positive neuronal cells. Cell nuclei were counterstained with Draq5. gcl, granule cell layer. Scale bars: **(B1)** 100 μm; **(D)** 25 μm; **(D1)** 5 μm. **(C2**,**D2)** Quantification of immunofluorescence signal reveals a significant reduction of KCC2 in the denervated oml/mml compared to the iml on the ipsilateral side, whereas no difference was found between the oml/mml and iml on the contralateral side. FI, fluorescence intensity. *N* (animals) = 7, *n* (ROIs) per layer = 42; Kruskal–Wallis test with Dunn’s multiple comparison test, *p ≤ 0.05.

To study KCC2 protein distribution, immunohistochemistry was performed in the dentate gyrus following entorhinal denervation. In the contralateral, i.e., non-lesioned dentate gyrus, a strong protein expression of KCC2 was detected in the oml/mml and inner molecular layer (iml) of the dentate gyrus 7 dpl (Figures 3A,A1,C,C1). In contrast, in the ipsilateral, i.e., lesioned DG, a strongly reduced immunoreactivity for KCC2 could be observed specifically in the denervated oml/mml, whereas KCC2 staining in the iml resembled the control situation (Figures 3B,B1,D,D1). Of note, strongly KCC2-expressing NeuN-positive neurons were found both in the non-denervated and denervated dentate oml/mml (Figures 3C,C1,D,D1). Quantification of immunofluorescence intensity confirmed the reduction in KCC2 protein signal in the denervated oml/mml (67.98 ± 8.45%) compared to the iml (100 ± 8.23%) on the ipsilateral side, whereas no difference was found between the oml/mml (100.8 ± 9.22%) and iml (100 ± 8.6%) on the contralateral side (Figures 3C2,D2).

### Entorhinal denervation leads to NKCC1 upregulation in reactive astrocytes in the outer and middle molecular layer of the dentate gyrus

**Figure 4:**
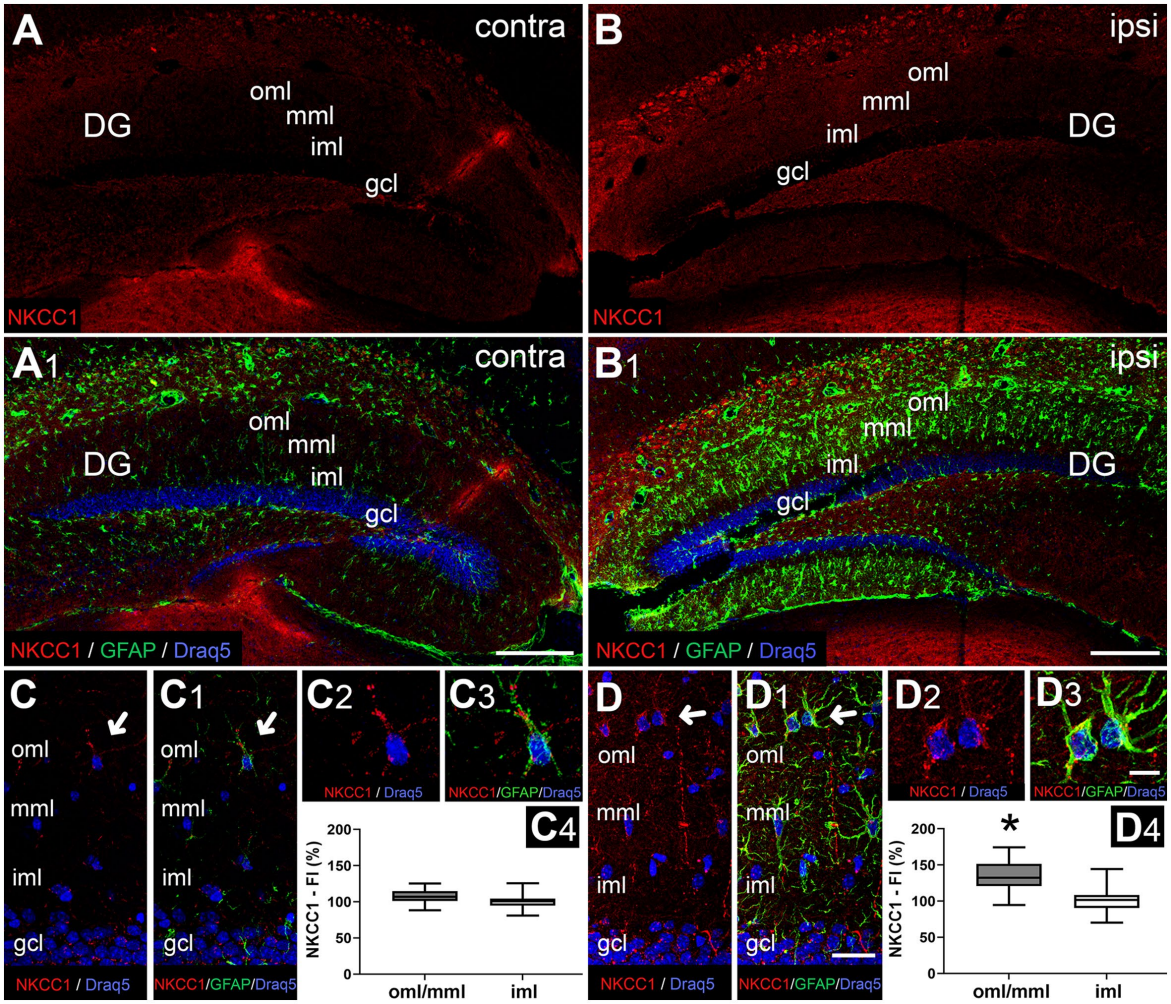
Entorhinal denervation leads to NKCC1 upregulation in reactive astrocytes in the outer and middle molecular layer of the dentate gyrus 7 days post-lesion. NKCC1 immunofluorescence of the dentate gyrus contralateral **(A,C)** and ipsilateral **(B,D)** to the lesion side reveals an upregulation of NKCC1 in the outer (oml) and middle (mml) molecular layer following entorhinal denervation. Immunostaining against glial fibrillary acid protein (GFAP) shows activation of glial cells, i.e., astrocytes, in the denervated oml/mml of the dentate gyrus **(B1,D1,D3)** compared to the contralateral side **(A1,C1,C3)**. Double-immunostained sections for GFAP and NKCC1 demonstrate increased NKCC1 expression in reactive astrocytes (arrows) in the ipsilateral **(B,D)** dentate gyrus compared to the contralateral side **(A,C)**. Higher magnification of NKCC1-positive astrocytes: **(C2,C3,D2,D3)**. Nuclei were counterstained with Draq5. gcl, granule cell layer; CA1, cornu ammonis region 1; CA3, cornu ammonis region 3. Scale bars: **(A1)** 150 μm; **(B1)** 150 μm; **(D1)** 25 μm; **(D3)** 10 μm. **(C4,D4)** Quantification of immunofluorescence signal reveals a significant increase of NKCC1 in the denervated oml/mml compared to the iml on the ipsilateral side, whereas no difference was found between the oml/mml and iml on the contralateral side. FI, fluorescence intensity. *N* (animals) = 7, *n* (ROIs) per layer = 42; Kruskal–Wallis test with Dunn’s multiple comparison test, *p ≤ 0.05.

In the non-lesioned (contralateral) dentate gyrus, only weak immunoreactivity for NKCC1 could be observed in the gcl and molecular layer of the dentate gyrus (Figures 4A,A1,C,C1–C3). In the denervated oml/mml, an increase in immunolabeling for NKCC1 compared to the control situation was found at 7 dpl, whereas an increase in the non-denervated iml was less apparent (Figures 4B,B1,D,D1–D3). The distribution pattern of NKCC1-positive cells in the oml/mml and gcl (Figure 4D) resembled that of reactive astrocytes seen after entorhinal denervation in mice (Del Turco et al., 2003) and, indeed, using a confocal double-labeling strategy, we could demonstrate that GFAP-positive astrocytes were strongly positive for NKCC1 in the denervated oml/mml (Figures 4C,D). Quantification of immunofluorescence intensity in combination with confocal analysis revealed a significant increase of NKCC1 signal in the denervated oml/mml (133.9 ± 19.75%) compared to the iml (100 ± 14.33%) on the ipsilateral side, whereas no difference was found between the oml/mml (106.7 ± 8.86%) and iml (100 ± 9.19%) on the contralateral side (Figures 4C4,D4).

## Discussion

**Figure 5:**
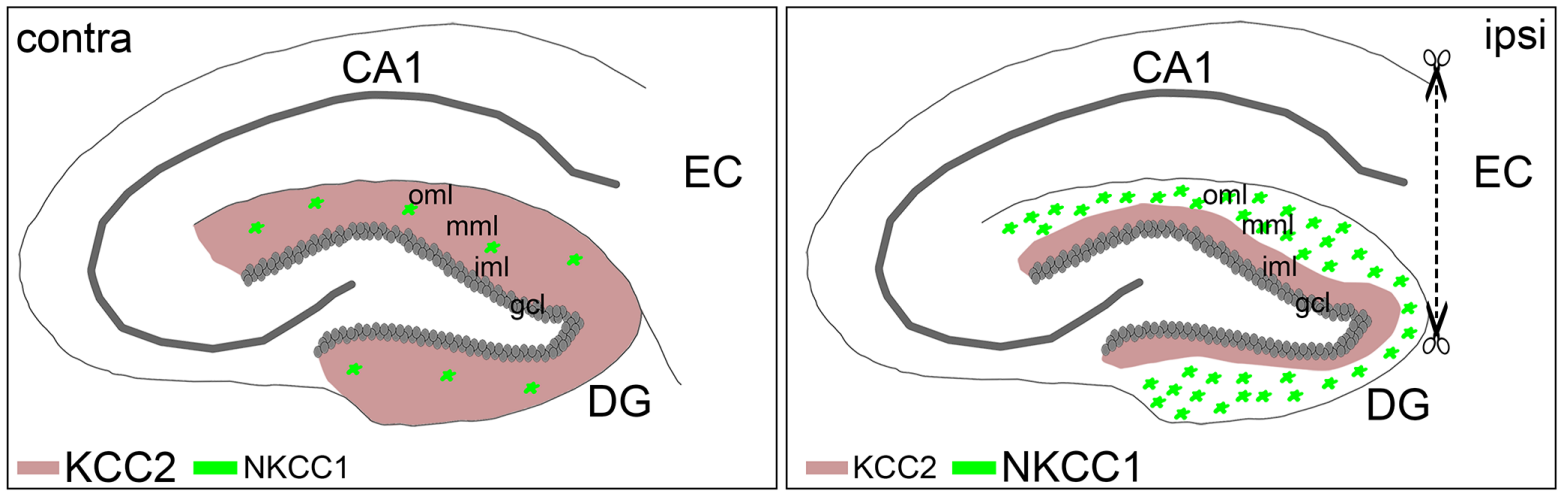
Schematic summary of KCC2 and NKCC1 expression changes following entorhinal denervation *in vivo*. Entorhinal denervation leads to layer-specific expression changes of KCC2 and NKCC1 in the dentate gyrus (DG): granule cells (gcl) downregulate *Kcc2* mRNA followed by decreased KCC2 protein expression in the denervated outer (oml) and middle (mml) molecular layer compared to the non-denervated inner molecular layer (iml), whereas reactive astrocytes, in contrast, upregulate *Nkcc1* mRNA as well as NKCC1 protein expression in the de-entorhinated zone following lesion (ipsi) compared to the control (contra) situation.

Entorhinal denervation is a well-established model for studying the neuronal reorganization processes following deafferentation (Deller and Frotscher, 1997; Deller et al., 2007; Perederiy and Westbrook, 2013). Using this model in combination with laser microdissection of defined cell layers, we detected cell- and layer-specific changes in the expression levels of the cation-chloride cotransporters KCC2 and NKCC1 following entorhinal denervation. Compared to non-denervated control mice, we found that *Kcc2* mRNA was strongly downregulated in the gcl of the dentate gyrus 7 dpl, while *Nkcc1* mRNA was upregulated in the oml/mml at this time point. Subsequent immunohistochemical staining revealed that KCC2 protein expression was reduced in the denervated oml/mml, where the dendrites of the granule cells are located, whereas NKCC1 protein was highly expressed in reactive astrocytes in the oml/mml of the denervated dentate gyrus. Together, these results suggest that KCC2 expression is selectively and transiently downregulated in granule cells while the increased NKCC1 expression is primarily mediated by reactive astrocytes in the denervated molecular layer (Figure 5).

The use of laser microdissection allowed us, for the first time, to pinpoint the expression changes of the two cation-chloride cotransporters in specific cell layers. Compared to controls, *Kcc2* mRNA was downregulated in the gcl. Since KCC2 is almost exclusively expressed in neurons (Kaila et al., 2014) and the cell bodies of the granule cells make up the majority of cells in the gcl, we concluded that *Kcc2* mRNA was selectively downregulated in granule cells. *In situ* hybridization in mice confirms that *Kcc2* mRNA is mainly confined to the gcl of the dentate gyrus (Allen Brain Atlas^2^). In contrast, KCC2 protein levels are highest in the molecular layer of the dentate gyrus, i.e., in the dendrites of the dentate granule cells, while the granule cell somata and axon initial segments show a comparatively weak expression (Gulyás et al., 2001; Báldi et al., 2010). The absence of *Kcc2* mRNA downregulation in the oml/mml at 7 dpl could indicate the presence of *Kcc2* mRNA in other cell types, such as inhibitory interneurons. Indeed, our immunostainings show that KCC2-expressing neurons are present in both the oml/mml of the denervated (ipsilateral) and the non-denervated (contralateral) hemispheres. However, compared to the ipsilateral iml and the contralateral oml/mml, the fluorescence intensity in the ipsilateral oml/mml was reduced, suggesting that KCC2 is primarily lost from the denervated dendritic segments of the granule cells but not from the denervated dendrites of interneurons. Interestingly, a recent study showed that a selective permanent loss of KCC2 in interneurons leads to cortical seizures due to alterations in the distribution of interneuron subtypes (Zavalin et al., 2022). That suggests that a granule cell-specific, transient reduction of KCC2 in the denervated dentate gyrus with preserved KCC2 in interneurons would not cause such a dramatic excitation-inhibition imbalance. Indeed, while electrolytic entorhinal cortex lesions were shown to induce hippocampal seizures (Dasheiff and McNamara, 1982; Kelley and Steward, 1996), the wire knife approach has not been associated with seizures (Dasheiff and McNamara, 1982). Taken together, our data show that entorhinal denervation leads to a reduction of *Kcc2* mRNA in granule cells as well as a selective downregulation of KCC2 protein expression in the denervated dendrites in the oml/mml.

The downregulation of KCC2 protein expression in the denervated granule cells would be expected to increase the intracellular Cl^−^ concentration and reverse the polarity of GABAergic currents, leading to an increase in neuronal excitability. KCC2 has been shown to be downregulated following different types of nervous system injury, such as chronic peripheral nerve constriction (Coull et al., 2003), spinal cord transection (Boulenguez et al., 2010), axotomy (Nabekura et al., 2002; Shulga et al., 2008), middle cerebral artery occlusion (Jaenisch et al., 2010), and deafferentation (Tighilet et al., 2016), indicating that the downregulation of KCC2 may be a common response to neuronal injury (Wu et al., 2016). Some authors have concluded that KCC2 downregulation is pathological, with depolarizing GABA responses promoting hyperexcitability (Boulenguez et al., 2010), excitotoxicity (Nabekura et al., 2002) and cell death (Kontou et al., 2021). Other authors have argued that reduced KCC2 expression may be beneficial by increasing the survival rate (Shulga et al., 2008) and promoting the reorganization of injured neurons (Jaenisch et al., 2010). It has even been speculated that the downregulation of KCC2 in the cochlear nucleus following unilateral cochlear neurectomy in cats may represent a homeostatic adaptation to the loss of synaptic input and preserve the activity levels in the deafferented neurons (Tighilet et al., 2016). Thus, the downregulation in KCC2 we observed in the denervated granule cells might reflect a homeostatic, rather than a pathological, response to deafferentation. However, depolarizing GABAergic inputs can also mediate inhibition by shunting excitatory inputs (Kilb, 2021). Therefore, it would be necessary to measure neuronal activity while monitoring KCC2 expression and chloride concentration in granule cells to determine whether KCC2 downregulation increases neuronal excitability. Since KCC2 agonists have been proposed for the treatment of spinal cord injuries (Hegarty and Stanicka, 2022), the transient downregulation of KCC2 in a denervated brain area suggests that treatment with KCC2 agonists could not only affect reorganization processes at injury sites but also brain rewiring in connected brain regions. Although currently hypothetical, our results and data from others (reviewed in Kaila et al., 2014; Hegarty and Stanicka, 2022) suggest that it may be worthwhile to investigate potential therapeutic effects.

Intriguingly, in addition to its chloride transport function, KCC2 protein has been shown to contribute to the regulation of excitatory and inhibitory synaptic function (Gauvain et al., 2011; Chamma et al., 2013; Chevy et al., 2015; Al Awabdh et al., 2022) and to the formation and maturation of spines (Li et al., 2007; Fiumelli et al., 2013; Awad et al., 2018). In line with this, KCC2 is expressed close to excitatory (Gulyás et al., 2001; Gauvain et al., 2011) and inhibitory synapses (Gulyás et al., 2001; Chamma et al., 2013). In this context, it is interesting to compare the time course of changes in spine density and KCC2 expression after denervation of the dentate gyrus. Entorhinal denervation leads to a transient loss of spines during the first week and a full recovery after several weeks (Parnavelas et al., 1974; Caceres and Steward, 1983; Vuksic et al., 2011). Therefore, it is tempting to speculate that the downregulation and recovery of KCC2 are associated with the denervation-induced reduction and recovery of spine density. However, future studies are needed to establish a mechanistic link between changes in KCC2 and spine remodeling triggered by entorhinal denervation.

In contrast to KCC2, both *Nkcc1* mRNA and NKCC1 protein expression were upregulated in the oml/mml following entorhinal denervation. Previous studies have similarly reported an increase in NKCC1 protein and/or mRNA levels in the brain and the spinal cord following injury (Yan et al., 2001a, 2018; Lu et al., 2008; Hui et al., 2016; Zhang et al., 2016). However, since NKCC1 is expressed in many different cell types (Kurki et al., 2022), we additionally performed immunohistochemical staining to validate the expression changes at the single-cell level. Based on the microarray and qPCR data showing a selective upregulation in the oml/mml, but not the gcl, we concluded that *Nkcc1* mRNA levels in the granule cells were not significantly affected by the lesion. That was consistent also with the expression of *Nkcc1* mRNA in the dentate gcl (Allen Brain Atlas^3^). Instead, we hypothesized that *Nkcc1* mRNA upregulation might be mediated by an increased expression in reactive astrocytes, which are induced by entorhinal denervation (Del Turco et al., 2003; Burbach et al., 2004). Therefore, we performed immunohistochemical staining for GFAP, which is upregulated in reactive astrocytes (Pekny and Pekna, 2014). The GFAP fluorescence was strongly increased in the denervated oml/mml and highly colocalized with NKCC1, which was also upregulated compared to the contralateral oml/mml. It was previously shown that reactive astrocytosis (quantified by GFAP immunofluorescence in the dentate gyrus) reaches a peak between 4 and 8 days and persists until about 12 days after aspiration of the entorhinal cortex in mice (Steward and Trimmer, 1997), which fits our microarray data showing a significant increase in *Nkcc1* mRNA between 3 and 14 dpl.

The increased expression of NKCC1 in reactive astrocytes might be related to a “dedifferentiation” to an immature, proliferative state (Buffo et al., 2008). It has been known for a long time that NKCC1 is involved in the regulation of cell proliferation in mouse fibroblasts (Panet et al., 2000), and inhibition of NKCC1 reduces the proliferation of mouse glioma cells (Luo et al., 2020). Following entorhinal cortex lesion in rats, the number of proliferating astrocytes is increased in the denervated cell layers (Hailer et al., 1999). However, increased NKCC1 expression in individual astrocytes and/or microglia, which also express functional NKCC1 (Tóth et al., 2022), could also account for the increased NKCC1 fluorescence in the oml/mml following entorhinal denervation. Interestingly, the electrophysiological characteristics of astrocytes also undergo a process of dedifferentiation following entorhinal cortex lesion in rats: the resting membrane potential and the input resistance are increased, which has been attributed to a decrease in inward-rectifying potassium current (Schröder et al., 1999). Buffering of the extracellular K^+^ concentration is an important astrocytic function, which is normally accomplished by the Na^+^/K^+^ pump and the inward-rectifying potassium channel Kir4.1 (Larsen et al., 2014). However, under pathological conditions, such as traumatic brain injury, NKCC1 activity or expression is upregulated and contributes to astrocytic swelling *in vitro* (Jayakumar et al., 2011) and *in vivo* (Zhang et al., 2016). Furthermore, NKCC1-deficient astrocytes do not swell in response to high extracellular K^+^ concentrations (Su et al., 2002), indicating that NKCC1 is essential for volume regulation during extreme conditions. Thus, it is possible that NKCC1 takes over the function of Kir4.1 in regulating the astrocytic volume and extracellular K^+^ concentration following entorhinal denervation, leading to an increased fluorescence signal. Understanding the cell-specific expression patterns of both KCC2 and NKCC1 following CNS and PNS injury is essential for developing effective treatments. For example, while several animal studies have reported that a blockade of NKCC1 upregulation following CNS injury improved clinical outcomes (Savardi et al., 2021), the situation appears to be different in the PNS. One study showed that increased activation of NKCC1 was necessary for the regeneration of myelinated sensory neurons in the dorsal root ganglion of rats that had undergone controlled sciatic nerve injury (Mòdol et al., 2015). Thus, NKCC1 upregulation may be pathological or beneficial depending on the cell type. Increasing KCC2 expression pharmacologically in mice with staggered spinal cord lesions improved the clinical outcome, but failed to do so in completely lesioned mice, indicating that the functional recovery was mediated by the reactivation of the remaining connections from the brain to the spinal cord that had been spared in the staggered lesion model (Chen et al., 2018). This reactivation of the spared circuitry was mainly accomplished by inhibitory interneurons and appears to be specific to KCC2 since increasing inhibition with GABA receptor agonists did not promote recovery (Chen et al., 2018). Therefore, it appears likely that some of the other functions of KCC2, such as the regulation of synaptic transmission, are even more important for the functional recovery after spinal cord injury than its chloride transport function.

In conclusion, we provide evidence for possibly homeostatic changes in KCC2 and NKCC1 expression in dentate granule cells and reactive astrocytes following entorhinal denervation *in vivo*. The temporary downregulation of KCC2 in the denervated granule cells might be associated with spine loss and contribute to a stabilization of the network activity by increasing GABAergic depolarization and neuronal excitability before other homeostatic mechanisms, such as dendritic retraction (Vuksic et al., 2011) and commissural sprouting by mossy cell axons (Del Turco et al., 2003) take effect, while the upregulation of NKCC1 in astrocytes might serve specific functions, such as enhancing proliferation and/or regulating the cell volume.

## Data availability statement

The original contributions presented in the study are included in the article/supplementary material, further inquiries can be directed to the corresponding author/s.

## Ethics statement

The animal study was reviewed and approved by Regierungspräsidium Darmstadt (Germany) and by the Ethics Committee of the University of Zagreb School of Medicine (Croatia).

## Author contributions

DD, JS, MP, JM, FB, and MV acquired and analyzed the data. DD, PJ, and TD conceived and supervised the study. All authors were involved in data interpretation, critically revising the manuscript, read, and approved the final manuscript.

## Funding

This work was supported by Deutsche Forschungsgemeinschaft (SFB 1080 to TD; DFG No. 467764793 and JE 528/10–1 to PJ).

## Conflict of interest

TD received a honorarium from Novartis for a lecture on human neuroanatomy.

The remaining authors declare that the research was conducted in the absence of any commercial or financial relationships that could be construed as a potential conflict of interest.

## Publisher’s note

All claims expressed in this article are solely those of the authors and do not necessarily represent those of their affiliated organizations, or those of the publisher, the editors and the reviewers. Any product that may be evaluated in this article, or claim that may be made by its manufacturer, is not guaranteed or endorsed by the publisher.

## Footnotes

^1^ https://imagej.net/software/fiji/

^2^ https://mouse.brain-map.org/gene/show/36415

^3^ https://mouse.brain-map.org/experiment/show/74882926
